# Presenteeism and psychosocial aspects of work among civil servants in leadership positions at a Brazilian federal university

**DOI:** 10.47626/1679-4435-2023-1126

**Published:** 2024-09-24

**Authors:** Letícia Paim Barbosa da Silva, Aline Silva-Costa

**Affiliations:** 1 Programa de Mestrado Profissional em Administração Pública em Rede, Universidade Federal do Triângulo Mineiro (UFTM), Uberaba, MG, Brazil; 2 Departamento de Saúde Coletiva, UFTM, Uberaba, MG, Brazil

**Keywords:** presenteeism, burnout, professional, occupational stress, public sector, leadership, presenteísmo, esgotamento profissional, estresse ocupacional, setor público, liderança

## Abstract

**Introduction:**

Presenteeism, defined as the physical presence of the worker in the work environment despite being sick, has been little investigated among public servants in leadership positions. Managers can suffer stress and even burnout, since the organizational environment is full of complex situations, which can generate strong tension.

**Objectives:**

To evaluate the relationship between work stress and burnout syndrome with presenteeism among civil servants in management positions at a Brazilian federal university.

**Methods:**

A total of 106 managers responded to an online questionnaire, including questions about the psychosocial aspects of work. Associations between presenteeism, sociodemographic aspects, dimensions of job stress (demand, control and social support) and Burnout syndrome (emotional exhaustion, dehumanization, emotional distancing and professional achievement) were performed using logistic regression methods.

**Results:**

In relation to presenteeism, 51.9% reported having worked in the presence of health problems, and 45.2% of presenteeist workers showed a drop in the performance of work activities due to presenteeism. Burnout syndrome was observed in 10.5% of the group. Regarding the stress at work, 42.9% showed high demand, 55.7% low control and 59.6% low social support. For presenteeists, associations were observed between decreased control, increased emotional exhaustion and dehumanization with greater chances of falling in work performance due to presenteeism.

**Conclusions:**

The characteristics of the management positions observed here are factors that can contribute to the greater occurrence of presenteeism and/or greater drop in work performance due to presenteeism.

## Abstract (pt)

**Introdução:**

A presença física do trabalhador no ambiente de trabalho, mesmo quando doente, definida como presenteísmo, tem sido pouco investigada entre servidores públicos em cargos de chefia. Os gestores podem sofrer estresse e *burnout,* visto que o ambiente organizacional é repleto de situações complexas que podem gerar forte tensão.

**Objetivos:**

Avaliar as relações do estresse no trabalho e da síndrome de *burnout* com o presenteísmo entre servidores nos cargos de gestão de uma universidade federal brasileira.

**Métodos:**

Estudo transversal com 106 gestores que responderam a um questionário on-line, incluindo perguntas sobre os aspectos psicossociais do trabalho. As associações bivariadas entre o presenteísmo, os aspectos sociodemográficos, as dimensões do estresse no trabalho (demanda, controle e apoio social) e da síndrome de *burnout* (exaustão emocional, desumanização, distanciamento emocional e realização profissional) foram realizadas por meio de análises de regressão logística binomial.

**Resultados:**

Do total, 51,9% dos participantes referiram ser presenteístas, sendo que 45,2% dos presenteístas apresentaram queda no desempenho das atividades laborais devido ao presenteísmo. A síndrome de *burnout* foi observada em 10,5% do grupo. Em relação ao estresse, 42,9% apresentaram alta demanda, 55,7% baixo controle e 59,6% apoio social baixo. Entre os presenteístas, foram observadas associações entre a diminuição do controle, o aumento da exaustão emocional e da desumanização com as maiores chances de queda no desempenho laboral devido ao presenteísmo.

**Conclusões:**

As características dos cargos de gestão aqui observadas são fatores que podem contribuir para a maior ocorrência de presenteísmo e/ou maior queda do desempenho laboral devido ao presenteísmo.

## INTRODUCTION

The physical presence of workers in the workplace, even in the face of a health problem, is defined as presenteeism. This phenomenon has been little explored in the literature, especially among civil servants in leadership positions. Presenteeism tends to reduce productivity and, given the difficulty of measuring presenteeism and its consequences on the work routine, the behavior tends to have a major negative effect on the work process and the worker’s health.^1^

Management and organizational mechanisms in the public service demand high productivity, imposition of goals, downsizing, and more flexible rights and pay. When civil servants responsible for running public services are absent from work, there are several consequences for the public administration, starting with the poor quality of services, which is detrimental to citizens.^2^

As for workers’ health, the changes that have been made to professional activity in recent decades have led to more stressful work activities.^3^ Conflicts with managers, poor social support at work, and barriers to dealing with problems are some examples of factors that tend to increase stress, with consequent damage to workplace dynamics, quality of life at work and health.^4^ A study on stress among civil servants working at a higher education institution found that the prevalence of stress was higher among professors who worked more than 40 hours a week and had worked at the institution for more than 8 years.^5^

In the literature, the organizational environment is full of complex situations that can generate a climate of strong stress among workers.^6^ When stressful situations are prolonged, they can lead to individuals suffering from burnout syndrome, a condition characterized by intense physical and mental exhaustion as a result of chronic work-related stress.^7^ According to the World Health Organization (WHO), “Burn-out is a syndrome conceptualized as resulting from chronic workplace stress that has not been successfully managed,” referring specifically to phenomena in the occupational context, and should not be applied to other life contexts.^8^ In January 2022, with the entry into force of the new International Statistical Classification of Diseases and Related Health Problems (ICD-11), the WHO recognized burnout syndrome as a work-related illness.

Therefore, understanding the psychosocial aspects of work related to presenteeism in the public service is directly linked to improving service provision for society. This is because this phenomenon is related to lower work quality and higher operating costs for institutions, due to the overload of workers and, above all, the impact on workers’ health and well-being.^9^

Gaining an understanding of the phenomenon of presenteeism, and the drop in productivity related to presenteeism contribute to discussions related to quality of life at work, helping to minimize the negative effects that stress has on individuals and organizations. This study aimed to evaluate the associations between two psychosocial aspects of work – dimensions of work stress and burnout syndrome – and presenteeism among civil servants in management positions at a Brazilian federal university.

## METHODS

The population eligible to participate in this study included civil servants in leadership positions, both tenured and deputy, at a federal university in Brazil. Of a total of 389 civil servants in leadership positions, 121 agreed to participate in the study. Once 15 participants’ forms had been excluded (7 participants filled out the form twice, one participant indicated on the Informed Consent Form [ICF] ‘do not agree to participate,’ and 7 participants did not fill in their name on the ICF), the final sample included 106 participants.

Data were collected by filling in a Google Forms online questionnaire and sending it to the civil servants by e-mail. Together with the online questionnaire, the ICF was presented. The questionnaire was made up of questions assessing sociodemographic aspects and general characteristics of civil servants’ work, including presenteeism, occupational stress, and burnout syndrome.

## STUDY VARIABLES

### Presenteeism

This variable was assessed by asking the following question: “In the last 30 days, have you been present at work despite having a health problem or any signs or symptoms of illness?” Servants who answered “yes” were classified as experiencing presenteeism.

### Lower work performance due to presenteeism

Workers experiencing presenteeism completed the Brazilian version of the Stanford Presenteeism Scale (SPS-6), which seeks to determine the worker’s ability to concentrate on work and complete activities despite health problems, and to identify how these problems interfere with workers’ productivity.^10^ The SPS-6 questionnaire, validated for use in Brazil, consists of six statements on a Likert-type scale, with five answer options ranging from 1: I strongly disagree with the statement to5:I strongly agree with the statement.^1^ Occupational performance due to presenteeism was assessed and the scores for the concentration dimension and the work completed dimension were totaled. The total points on the scale range from 6 to 30. Lower scores (6 to 18) indicate lower performance in work activities and less concentration. On the other hand, higher scores (19 to 30) indicate that workers’ health conditions have less influence on their activities, corresponding to a greater ability to concentrate and perform their work despite their health problems.^1,11^

### Reasons for presenteeism

This was assessed using the question “What led you to work despite having any signs/symptoms of illness?”

### Occupational stress

This was assessed using the dimensions of the Job Stress Scale (JSS), with 17 questions: five to assess work demands, six to assess control and six for social support, adapted for Brazilian Portuguese.^12^ The answer options for the demand and control dimensions are presented on a Likert scale (1-4), ranging from “often” to “never/almost never.” The answers for the social support dimension are presented on a Likert scale (1-4) ranging from “strongly agree” to “strongly disagree.”^12^ Scores above the median represented high demand, high control, and high social support, respectively.

### Burnout syndrome

This is assessed using Part II of the *Inventário para Avaliação da Síndrome de Burnout* (ISB, Inventory for the Assessment of Burnout Syndrome), an instrument developed and validated in Brazil.^13^ Part II of the ISB consists of 19 items subdivided into 4 scales: emotional exhaustion (EE) with 5 items, dehumanization (DEs) with 5 items, emotional detachment (DEm) with 4 items and professional fulfillment (RP) with 5 items.^14,15^ The items comprise a 5-point Likert scale, ranging from 0 (never) to 4 (every day).^15^ The mean scores are 4 to 9 for EE, 10 to 15 for RP, 2 to 6 for DEs and 4 to 7 for DEm. Scores above 9 for EE, 6 for DEs, and 7 for DEm suggest problems. For PR, scores below 10 indicate problems. People who scored high EE and high DEs and/or DEm and low RP were considered to have burnout.^16^

## COVARIATES

The questionnaire also included the following variables to characterize the group interviewed: age (categorized as a median: up to 38 years; 39 years or more), sex (female; male), marital status (married/stable union; single/separated/divorced/widowed), education (college degree/specialization; master’s/doctorate), management positions (tenured; deputy), working hours (up to 40 h/week; more than 40 h/week), length of service at the university (categorized as a median: up to 8 years; more than 8 years) and length of service in the management position (up to 3 years; more than 3 years).

## DATA ANALYSIS

We conducted descriptive analyses, expressed in absolute and relative frequencies, to characterize the study population. Bivariate associations of presenteeism with sociodemographic aspects, the dimensions of work stress (demand, control, and social support) and burnout syndrome (EE, DEs, DEm, and RP), and the other occupational factors were analyzed using Pearson’s chi-square test.

Multiple binomial logistic regression analyses investigated the associations between the dimensions of occupational stress and burnout syndrome and presenteeism. These analyses included social support, considering the literature on the possible moderating role of this factor in the relationship between psychosocial aspects of work and health.^17,18,19^ Variables with p ≤ 0.20 in the bivariate associations were included in the multiple regression model. The associations between the dimensions of occupational stress and burnout syndrome and the drop in work performance due to presenteeism were also tested using multiple binomial logistic regression models. The results were expressed as odds ratios (OR) and 95% confidence intervals (95%CI). A significance level of 5% was considered and RStudio version 4.1.0 was used.

The Research Ethics Committee of the institution approved the study (Certificate of Submission for Ethical Appraisal: 31852620900005154). All the civil servants who agreed to participate in the study signed an ICF.

## RESULTS

Most civil servants in leadership positions were women (53.3%), 52.8% were up to 38 years old and 67.9% were married. All had a college degree, and 74.5% had a master’s or doctorate. As for their job history, 51.9% held only one permanent position, 59.4% worked more than 40 hours a week, 56.2% worked at the university for 8 years or less and 47.2% held leadership positions for more than 3 years. Burnout syndrome was present in 10.5% of the group: 39.1% had high EE, 51.1% had low RP, 23.3% had high DEm, and 53.5% had high DEs. In relation to the dimensions of stress at work, 42.9% of the group had high demands, 55.7% had low control, and 59.6% had low social support.

Meanwhile, 51.9% of the group reported working even though they had health problems, and 45.2% of those who reported presenteeism showed a drop in their work performance. Statistically significant associations were found between presenteeism and work demand (p = 0.034), DEm (p = 0.013) and EE (p = 0.055). There was a higher frequency of presenteeism among employees with high demand (64.4%), high DEm (70.8%) and high EE (65.8%) (Table 1).

**Table 1:** Sociodemographic and work characteristics of civil servants in leadership positions at a federal university in Brazil in 2021

Variables	Presenteeism n (%)		p value
	No 51 (48.1)	Yes 55 (51.9)	
Age			
Up to 38 years	25 (44.6)	31 (55.4)	0.574
Over 38 years	26 (52.0)	24 (48.0)	
Sex*			
Female	25 (44.6)	31 (55.4)	0.506
Male	26 (53.1)	23 (46.9)	
Marital status			
Married/stable union	36 (50.0)	36 (50.0)	0.721
Single/separated/widowed	15 (44.1)	19 (55.9)	
Education			
College degree/specialization	14 (51.9)	13 (48.1)	0.820
Master’s/Doctorate	37 (46.8)	42 (53.2)	
Leadership positions			
Tenured	30 (54.5)	25 (45.5)	0.371
Deputy	11 (39.3)	17 (60.7)	
Tenured and deputy	10 (43.5)	13 (56.5)	
Working hours			
Up to 40 h/week	22 (51.2)	21 (48.8)	0.748
Over 40 h/week	29 (46.0)	34 (54.0)	
Length of service at UFTM†			
Up to 8 years	29 (49.2)	30 (50.8)	0.999
More than 8 years	22 (47.8)	24 (52.2)	
Time in a leadership position			
Up to 3 years	28 (50.0)	28 (50.0)	0.828
More than 3 years	23 (46.0)	27 (54.0)	
Workload			
Low	35 (58.3)	25 (41.7)	0.034
High	16 (35.6)	29 (64.4)	
Control at work			
High	22 (46.8)	25 (53.2)	0.965
Low	29 (49.2)	30 (50.8)	
Social support at work			
High	23 (54.8)	19 (45.2)	0.356
Low	27 (43.5)	35 (56.5)	
Burnout syndrome			
No	48 (51.1)	46 (48.9)	0.081
Yes	2 (18.2)	9 (81.8)	
Emotional exhaustion			
Low	13 (61.9)	8 (38.1)	0.055
Medium	24 (55.8)	19 (44.2)	
High	14 (34.2)	27 (65.8)	
Professional fulfillment			
Low	20 (41.7)	28 (58.3)	0.590
Medium	16 (51.6)	15 (48.4)	
High	8 (53.3)	7 (46.7)	
Emotional detachment			
Low	32 (62.7)	19 (37.3)	0.013
Medium	11 (39.3)	17 (60.7)	
High	7 (29.2)	17 (70.8)	
Dehumanization level			
Low	6 (50.0)	6 (50.0)	0.205
Medium	21 (60.0)	14 (40.0)	
High	22 (40.7)	32 (59.3)	

* One participant marked “other”.

† Missing data (n = 1).

The reasons civil servants in leadership positions mentioned for staying at work even when ill were divided into organizational and individual factors, as shown in Table 2.

**Table 2:** Reasons that led civil servants in leadership positions at a federal university in Brazil to engage in presenteeism in 2021

Organizational factors	Individual factors
Need for the job	Commitment/responsibility/fulfilling their role
Deadlines	Their desire to get the job done
Bureaucracy	Honor their position
Institutional, urgent demands/pressure	The possibility to reconcile rest with working from home
Accumulated work/not to accumulate work	Distraction from symptom/illness
Not to have a deputy/have to do other people’s work	Mild symptoms/symptoms that do not interfere with doing the job
Demands, text/voice messages, calls on apps	Enjoy what they do
Requests from students/advice for students	Not to overload colleagues/help colleagues
No trained person to do the job	Perform the necessary duties for running the department smoothly

Table 3 shows the associations between psychosocial aspects of work and presenteeism, according to the degree of perceived social support. It was found that among civil servants with low social support, for each additional point on the DEm scale, the chance of presenteeism increased by 47% (OR = 1.47; 95%CI 1.17-1.97). For DEs, each additional point on the scale was associated with a 26% decrease in the chance of presenteeism (OR = 0.74; 95%CI 0.58-0.91). Among employees with high social support, an increase in demand scores was associated with a 46% greater chance of presenteeism (OR = 1.46; 95%CI 1.04-2.22).

**Table 3:** Association between psychosocial aspects of work and presenteeism among civil servants in leadership positions at a federal university in Brazil in 2021

	Low social support	High social support
	Presenteeism OR (95%CI)	
Demand	1.35 (0.89-1.90)	1.46 (1.04-2.22)*
Control		
Emotional exhaustion	1.12 (0.93-1.36)	0.97 (0.82-1.16)
Emotional detachment	1.47 (1.17-1.97)*	0.88 (0.61-1.20)
Dehumanization	0.74 (0.58-0.91)*	1.18 (0.88-1.62)

Explanatory variables analyzed using the total score. Models stratified by social support and adjusted for age and working hours. OR = odds ratio; CI = confidence interval.

* Statistically significant associations resulting from the binomial logistic regression model.

A statistically significant association was observed between lower control at work and a 38% increase in the drop in performance due to presenteeism (OR = 0.62;95%CI 0.41-0.88). In addition, statistically significant associations were observed between EE, DEs and performance decline due to presenteeism. The chances of a drop in performance were 21% higher as EE and DEs scores increased (Table 4).

**Table 4:** Association between work stress - demand, control, and social support -, burnout syndrome - emotional exhaustion, dehumanization, and emotional detachment -and performance decline due to presenteeism among civil servants in leadership positions at a federal university in Brazil in 2021

	Performance decline due to presenteeism OR (95%CI)
Demand	1.03 (0.78-1.38)
Control	0.62 (0.41-0.88)*
Social support	0.88 (0.70-1.08)
Emotional exhaustion	1.21 (1.05-1.42)*
Emotional detachment	0.78 (0.59-1.00)
Dehumanization	1.21 (1.01-1.48)*

Explanatory variables analyzed using the total score. Model adjusted for age and working hours. OR = odds ratio; CI = confidence interval.

* Statistically significant associations resulting from the binomial logistic regression model.

## DISCUSSION

The sample of civil servants in leadership positions at a federal university in Brazil is characterized by managers who mostly work more than 40 hours a week, have low control over their work, low social support and have experienced presenteeism. The associations between presenteeism and the dimensions of work stress and burnout syndrome, with different results depending on the degree of social support (high or low), reinforce the moderating role of this factor in the associations observed. In the case of the sub-sample of presenteeism, the relationship between decreased control, and increased EE and DEs with greater chances of performance decline in work activities due to presenteeism highlights the potential harm of presenteeism for both the worker and the employer.

The strong association between presenteeism and high work demand observed in this study may be due to short time to meet work demands. Hansen & Andersen^20^ found that workers, after being absent for some reason, may find that on returning to work they face even higher demands due to accumulated tasks resulting from their absence. This relationship can lead to workers choosing to remain at work even if they are ill, in the hope of meeting their demands. Factors such as fear of not being seen as committed to the job, fear of overloading colleagues, concern about delays in their work and fear of services not being provided to clients or the community are some of the reasons why workers go to work despite being ill.^21^ As observed in this study, presenteeism would be a characteristic of loyalty, motivation, and a sense of responsibility.^21^

Although there were significant relationships between demand and presenteeism from the perspective of stress at work, no significant associations were found between the control dimension and presenteeism. Similarly, other authors found no such association.^20,22,23^ The reason for not finding a relationship can be attributed to the difficulty of establishing a specific direction for the relationship between presenteeism and control over work. On the one hand, workers with high control may adjust their demands to their physical and mental capacity and thus increase their presenteeism. On the other hand, workers with low control may have little autonomy to reorganize work tasks, which can contribute to presenteeism.^24^ Although greater control gives workers more autonomy and freedom to do their job, it can also mean greater pressure and responsibility, which can end up negatively affecting their health^25^ and their work. The result observed for the relationship between a reduction in control and a greater chance of poor performance at work due to presenteeism stands out in this respect.

Some authors believe that social support is a mediator in the demand-control relationship. Social support reduces the negative effects of high demands and low control, increasing feelings of well-being. On the other hand, the absence of social support can aggravate the negative effects on the worker.^21^ According to Johnson & Hall,^26^ poor social support seems to accentuate the impact of work stress, since workers with low support had higher prevalence of cardiovascular diseases at every level of work stress.

As for the moderating role of social support, in which high demand was associated with presenteeism only among managers with a high level of support, Saijo et al.^17^ point out that when social support is high, workers find it easier to take time off, since they can rely on their colleagues for help. A study of technical-administrative staff at a higher education institution, on the other hand, found no significant association between the role of social support in the relationship between work demands and presenteeism. However, in the case of a sample of workers in leadership positions, such as the one represented in this study, good relations between workers may lead to greater commitment on the part of the manager, leading them to embrace presenteeism when faced with high demands, with a view to not harming the team.

As to the relationships between the dimensions of burnout and presenteeism, the associations between higher DEm, lower DEs and higher chances of presenteeism only among managers with low social support are also based on the role of social support in working relationships. A meta-analysis identified statistically significant relationships between low social support and common mental disorders.^27^ From a similar perspective, a study of federal civil servants found that interpersonal difficulties were positively related to EE and depersonalization, showing that low social support in the workplace was associated with higher levels of emotional exhaustion and detachment from clients.^28^ Another study found that EE, low PR and depersonalization positively predicted presenteeism.^29^

Demerouti et al.^30^ studied the relationship between work demands, the dimensions of burnout (exhaustion and depersonalization), and presenteeism, and found that work demands are more frequently associated with presenteeism, whereas depersonalization is a consequence of presenteeism over time. The authors also found that exhaustion and presenteeism are reciprocal, because when workers feel exhausted, they develop strategies to compensate for their performance and because they exhibit presenteeism, their exhaustion tends to increase over time. As they do not take time off to look after their health, presenteeism impairs their recovery, causing them to develop negative attitudes towards their work and thus develop depersonalization.^30^ These findings^30^ may help to explain the relationship between the increase in EE and DEs and the greater chances of a decline in work performance due to presenteeism.

The relationship between presenteeism and psychosocial factors at work can be investigated from different directions or even from a bidirectional perspective. Presenteeism can precede physical and mental exhaustion, burnout syndrome, stress, and depression. Workers find themselves trapped in a vicious circle, in which high demand can make them work even if they show some sign/symptom or condition. At the same time, presenteeism can cause the worker to have less energy to deal with the demands, causing signs of burnout.^21^

This study has the strength of having been conducted with civil servants in leadership positions, a population for which the literature is still scarce. In addition, the fact that the study jointly addressed presenteeism and its relationship with the dimensions of the Demand-Control Model and burnout syndrome contributes to a knowledge gap, which is the identification of psychosocial factors at work, considering the particularities of public institution management. Although no statistically significant associations were found between burnout syndrome and presenteeism in this study, the assessment of burnout using the dimensions DEs, DEm, EE, and RP showed the extent to which each of these constructs separately can interact with presenteeism.

Some limitations are also worth pointing out. Firstly, the study was conducted during the COVID-19 pandemic, which prevented the questionnaires from being completed in person at the workplace of each civil servant. This may have influenced some responses, since managers were working from home and not in their usual work environment. Another limitation of this study is that all civil servants were evaluated together, and not according to the type of position held, as some positions may have specific and different characteristics to other managerial positions. We therefore suggest that future studies analyze management positions separately: incumbents and deputies and/or teaching staff and technical-administrative staff. Finally, given that there has been little research into public servants, especially managers, the comparison between studies based on the same population group has been limited.

In conclusion, this study has shown that the characteristics of leadership positions observed here, namely high demands, low control over work, low social support, high EE, high DEs and low RP are factors that can contribute to a greater prevalence of presenteeism and/or a greater drop in work performance due to presenteeism.
